# Characterization of In Vitro Performance and Hemocompatibility of a Novel Hemofilter for Continuous Kidney Replacement Therapy

**DOI:** 10.3390/membranes16100338

**Published:** 2026-10-12

**Authors:** Adam M. Zawada, Lukas Theis, Robert Nitschel, Karlee Emal, Robert Pohlmeier, Skyler Boyington, Ansgar Erlenkoetter

**Affiliations:** 1 Product Development, Fresenius Medical Care Deutschland GmbH, 66606 Sankt Wendel, Germany; 2 Biosciences, Fresenius Medical Care Deutschland GmbH, 66606 Sankt Wendel, Germany; robert.nitschel@freseniusmedicalcare.com (R.N.); ansgar.erlenkoetter@freseniusmedicalcare.com (A.E.); 3 Applications Laboratory, Fresenius Medical Care, Ogden, UT 84404, USA; karlee.emal@freseniusmedicalcare.com (K.E.); skyler.boyington@freseniusmedicalcare.com (S.B.); 4 Medical Education and Information EMEA, Fresenius Medical Care Deutschland GmbH, 61352 Bad Homburg, Germany; robert.pohlmeier@freseniusmedicalcare.com

**Keywords:** hemofilter, membrane, hydrophilicity, performance, hemocompatibility, CKRT

## Abstract

Performance and hemocompatibility are the main focus areas in the development of new hemofilters for continuous kidney replacement therapy. The novel multiFlux hemofilter features a polysulfone membrane with increased hydrophilicity to improve performance stability and hemocompatibility. In the present in vitro study, we compared the multiFlux hemofilter to established hemofilters on the market, i.e., Ultraflux AV (polysulfone-based), Prismaflex HF (polyarylethersulfone-based) and Prismaflex ST (AN69 ST-based). For the characterization of performance, dialysate-side plasma clearance of urea, creatinine and β2-microglobulin (β2M) were investigated over 24 h. Hemocompatibility was characterized in a blood recirculation setup by determining activation of platelets, plasmatic coagulation, the complement system and leukocytes. For urea and creatinine clearance, all hemofilters reached or approached the effluent limit across all time points. For β2M, the multiFlux hemofilter showed significantly higher clearance values at each time point vs. all comparators (*p* < 0.001) and the most stable performance over 24 h. The multiFlux hemofilter also showed a favorable hemocompatibility profile, especially in terms of platelet activation (vs. Ultraflux AV and Prismaflex HF) and complement activation (vs. all comparators). This in vitro study supports previous findings that increased membrane hydrophilicity improves performance stability and hemocompatibility of synthetic membranes.

## 1. Introduction

Acute kidney injury is a complex syndrome including a typically rapid decline in renal function [1,2,3], affecting over 50% of patients admitted to the intensive care unit [4]. Besides the management of the underlying disorder, continuous kidney replacement therapy (CKRT) is a cornerstone in the treatment of severe acute kidney injury, providing continuous solute clearance and fluid regulation [5,6].

The hemofilter represents a central component of each CKRT system, with performance and hemocompatibility being key determinants of safe and efficient treatments. Here, the membrane of the hemofilter is the main determinant of both performance and hemocompatibility, given its large surface area, selective permeability and direct interaction with the patient’s blood. Blood–membrane interactions may trigger undesired effects, including coagulation, complement and leukocyte activation, and may compromise both hemofilter function and patient safety [7,8,9,10,11]. Moreover, adsorption of proteins to the membrane over treatment time may alter membrane permeability, thereby affecting clearance of uremic toxins, particularly of larger molecules such as β2-microglobulin [12,13,14,15,16].

Continuous development of hemofilter membranes over recent decades has improved toxin removal capacity and hemocompatibility as compared to earlier cellulose-based membranes [17,18,19,20]. Synthetic membranes, typically composed of polysulfone or polyethersulfone, are now the standard in hemodialysis. However, variations in manufacturing and sterilization processes result in significant differences in membrane properties, including fiber geometry; undulation; packing density; pore size; surface charge; and, in particular, membrane hydrophilicity. Given the hydrophobic characteristics of poly(arlyether)sulfone, polyvinylpyrrolidone (PVP) is typically added to the spinning mass as a hydrophilic agent. Increased membrane hydrophilicity reduces membrane fouling and is associated with improved hemocompatibility and more stable performance over treatment time [12,21,22,23,24,25,26,27,28,29]. This is of particular importance for CKRT, given the prolonged treatment duration with a single hemofilter and the lower blood flow rates compared to intermittent treatments of patients with chronic kidney disease, which typically last about 4 h and use higher blood flow rates.

The novel multiFlux hemofilter for CKRT uses the technology of the Helixone *hydro* membrane, which was previously shown to feature increased PVP content and hydrophilicity, associated with lower membrane fouling [28,30]. To investigate the Helixone *hydro* membrane in the context of CKRT, in the present study, the multiFlux hemofilter was compared to other established hemofilters on the CKRT market in terms of hemocompatibility and performance.

## 2. Materials and Methods

### 2.1. Investigated Hemofilters

The novel multiFlux hemofilter (Fresenius Medical Care, Bad Homburg, Germany) was compared to three established hemofilters on the market with polysulfone (Ultraflux AV, Fresenius Medical Care, Bad Homburg, Germany), Polyarylethersulfone (Prismaflex HF, Baxter/Vantive, Deerfield, USA) and AN69 ST (Prismaflex ST, Baxter/Vantive, Deerfield, USA) membranes, respectively. Further information regarding the investigated hemofilters is presented in Table 1.

### 2.2. Investigation of Performance over Time

Performance of the hemofilters was investigated over 24 h in a two-step approach, with (1) determination of urea (60 Da), creatinine (113 Da) and β2-microglobulin (β2M, 12 kDa) dialysate-side plasma clearance at different time points and (2) protein fouling in a plasma recirculation setup to induce membrane fouling over 24 h. An overview of the setup is presented in Figure 1.

#### 2.2.1. Determination of Plasma Clearance

To characterize long-term performance of the hemofilters, dialysate-side plasma clearance of urea, creatinine and β2M was determined at different time points of plasma recirculation (0, 2, 4, 8, 16, and 24 h; see Section 2.2.2). The hemofilters were first filled and de-aerated. The multiFlux, Ultraflux AV and Prismaflex HF hemofilters were filled and de-aerated using an in-house prepared acetate solution, whereas the Prismaflex ST150 hemofilter was filled and de-aerated with a heparin spiked isotonic sodium chloride solution (Fresenius Medical Care), in accordance with the manufacturer’s instruction for use. For each time point of clearance measurement, 1.2 L of bovine plasma (37 °C, 6.0 ± 0.5 g/dL total protein concentration, 1% sodium heparin, Lampire Biological Laboratories, Pipersville, USA) was supplemented with 2.25 mg/L β2M (Medix Biochemica, Maryland Heights, USA), 150 mg/L creatinine (Sigma-Aldrich, St. Louis, USA), and 1.9 g/L urea (Sigma-Aldrich). The measurement was performed in a single-pass mode with the following flow conditions: Blood-side flow rate of Q_B_ = 100 mL/min, dialysate flow rate of Q_D_ = 33 mL/min (acetate based dialysate) and filtrate flow rate of Q_F_ = 17 mL/min. Samples were obtained at the blood inlet and dialysate outlet, and the concentrations of the solutes were determined with an immunoturbidimetric assay for clinical chemistry analyzers (Roche/Hitachi, Indianapolis, USA) for β2M, an enzymatic creatininase clinical chemistry assay (Roche/Hitachi) for creatinine, and a kinetic blood urea nitrogen clinical chemistry assay (Roche/Hitachi) for urea. For each solute, technical triplicates were analyzed, and the means for each measurement were determined.

The following formula was used to calculate the dialysate-side clearance:

$$Clearance=\frac{Concentration\;dialysate}{Concentration\;blood\;inlet}\;\times \;(Dialysate\;flow\;rate+\;Filtrate\;flow\;rate)$$

For each hemofilter type, three independent performance experiments were performed with predefined test points over 24 h of plasma recirculation. The data of the three experiments are presented as means ± SD (standard deviation) and compared by using one-way ANOVA with Dunnett’s post hoc test at each time point.

#### 2.2.2. Plasma Recirculation to Induce Membrane Fouling

For the protein adsorption step, 3 L of bovine plasma (37 °C, 6.0 ± 0.5 g/dL total protein concentration, 1% sodium heparin, Lampire Biological Laboratories), supplemented with 6.7 IU/mL sodium heparin (Fresenius Kabi, Lake Zurich, USA), was recirculated with a plasma flow rate of Q_B_ = 100 mL/min, dialysate flow rate of Q_D_ = 33 mL/min and filtrate flow rate of Q_F_ = 17 mL/min. The ultrafiltrate was fully substituted with normal saline provided under the same flow condition (17 mL/min). Every 4 h, the system was briefly paused to exchange the plasma reservoir without introducing air into the circuit. The total recirculation time was 24 h, with additional pausing during the clearance measurements at the predefined time points (Section 2.2.1 and Figure 1).

### 2.3. Characterization of Hemofilter Hemocompatibility

The hemocompatibility setup was recently described in detail [31]. In brief, testing was performed using 450 mL of whole blood from healthy donors, participating in the company’s blood donor program. All donors provided written informed consent, and the blood samples were anonymized prior to use. Blood was collected using a 17 G dialysis needle (Fresenius Medical Care) and was supplemented with 750 IU heparin (Ratiopharm GmbH, Ulm, Germany) diluted in 50 mL saline solution (Serumwerk Bernburg Vertriebs GmbH, Bernburg, Germany; final heparin concentration: 1.5 IU/mL). The blood was distributed between two parallel recirculation setups to enable a direct comparison between the multiFlux and a comparator hemofilter (i.e., ~220 mL of blood per recirculation loop), thereby reducing inter-donor variability (Figure 2). Before filling with blood, both recirculation loops were placed in the same incubator (37 °C; Memmert, Schwabach, Germany), and the systems were rinsed with saline solution for ~30 min. The recirculation was performed over 180 min without dialysate flow and with a blood flow rate of 200 mL/min. Blood samples were taken before recirculation (0 min) as well as at 15, 30, 60, 120 and 180 min during blood recirculation.

The blood samples were used to characterize platelet activation (platelet loss, β-thromboglobulin [β-TG]), plasmatic coagulation activation (thrombin–antithrombin III complex [TAT], activated clotting time [ACT]), complement activation (complement factor sC5b-9), and leukocyte activation (polymorphonuclear neutrophil [PMN] elastase). Platelet counts were determined with an automatic hematology analyzer (XN-1000 Pure, Sysmex, Kobe, Japan). β-TG (Asserachrom β TG, Diagnostica Stago, Asnières-sur-Seine, France), TAT (Enzygnost TAT micro, Siemens Healthineers AG, Forchheim, Germany), sC5b-9 (MicroVueTM sC5b-9 Plus EIA, QuidelOrtho, San Diego, CA, USA) and PMN elastase values (Human PMN-Elastase, Thermo Fisher Scientific Inc., Dreieich, Germany) were measured with ELISA (enzyme-linked immunosorbent assay) kits, and ACT was determined with an automatic coagulation timer system (low-range activated clotting time, ACT Plus^®^, Medtronic GmbH, Meerbusch, Germany). Two technical replicates were performed for the measurement of platelet counts and ACT, and three technical replicates were performed for all ELISA measurements.

To evaluate hemocompatibility markers, the respective area under the curve (AUC) was determined for each parameter, after hematocrit correction (platelets: corrected value [t] = value [t] × hematocrit [t = 0]/hematocrit [t]; plasma markers: corrected value [t] = value [t] × hematocrit [t = 0]/hematocrit [t] × (100% − hematocrit[t])/(100% − hematocrit [t = 0]). For platelet loss, the AUC up to 60 min was calculated to capture the platelet dynamics in this time range, and the percentage decrease was determined based on the AUC per minute relative to t = 0 min levels. For β-TG, TAT, sC5b-9 and PMN elastase, the AUC was determined over 180 min, normalized based on t = 0 min concentrations and presented as hourly increase for each marker. For ACT, the decrease between 15 and 180 min was determined and compared between the multiFlux and the comparator hemofilters.

Six pairwise comparisons were performed for each comparator hemofilter vs. multiFlux. The data are presented as means ± SD and compared using the paired Student’s *t*-test.

## 3. Results

### 3.1. Characterization of Hemofilter Performance over Time

Dialysate-side plasma clearances of urea, creatinine and β2M were determined at multiple time points during the 24 h of plasma recirculation (time points: 0, 2, 4, 8, 16, and 24 h). The results are summarized in Figure 3 and Supplementary Table S1.

For urea clearance, all hemofilters reached or approached the operational limit by the effluent flow (Q_D_ + Q_F_) across all time points. For some timepoints, the multiFlux hemofilter showed significantly higher urea clearance values (0 h: vs. Ultraflux AV; 2 h: vs. Prismaflex ST; 4 h: vs. Ultraflux AV and Prismaflex ST). Moreover, the performance remained stable throughout the experiments for all time points and hemofilters, except for the Prismaflex ST hemofilter showing a slight but not significant decrease at 24 h (8.6% vs. 0 h, *p* = 0.161).

Comparable results were observed for the creatinine clearance, with all hemofilters reaching or approaching the operational limit by the effluent flow. For some time points, the multiFlux hemofilter showed significantly higher creatinine clearance values (0 h: vs. Ultraflux AV and Prismaflex ST; 4 h: vs. Ultraflux AV and Prismaflex ST; 16 h: vs. Ultraflux AV and Prismaflex ST). Moreover, the performance remained stable throughout the experiments for all time points and hemofilters, except for the Prismaflex ST hemofilter showing a slight but not significant decrease at 24 h (12.6% vs. 0 h, *p* = 0.138).

Clear differences between the four hemofilters were observed for the larger β2M molecule, with significantly higher values at each time point for the multiFlux hemofilter vs. all comparators (*p* < 0.001 for all time points and comparisons). Due to some small differences in the membrane surface size (multiFlux: ± 0.2 m^2^ vs. comparators), clearance values were also analyzed after surface normalization (Supplementary Figure S1). This analysis also showed that the multiFlux hemofilter had significantly higher clearance values vs. all comparators and across all timepoints. Moreover, the β2M clearance remained mostly stable over 24 h for the multiFlux hemofilter (8.2% decrease, *p* = 0.181 vs. 0 h), followed by the Prismaflex HF (40.4%, *p* < 0.001), the Ultraflux AV (44.0%, *p* < 0.01) and the Prismaflex ST (50.8%, *p* < 0.05) hemofilters.

It is important to note that the Prismaflex ST hemofilter showed a different pattern of dialysate-side β2M plasma clearance due to the strong absorption effects of the AN69 ST membrane, as also reported in other studies [31,32]. Therefore, this low dialysate-side clearance cannot be equated with low β2M removal.

### 3.2. Characterization of Hemofilter Hemocompatibility

To characterize the hemocompatibility of the hemofilters, four different categories were investigated in the following sections: platelet activation (Section 3.2.1), plasmatic coagulation activation (Section 3.2.2) and complement and leukocyte activation (summarized in Section 3.2.3).

#### 3.2.1. Platelet Activation

For platelet activation, platelet loss and β-TG increase over time were investigated across the four hemofilters. The results of the pairwise comparisons vs. the multiFlux hemofilter are summarized in Figure 4 and Supplementary Table S2.

The multiFlux hemofilter showed significantly lower platelet loss as compared to the Ultraflux AV (13.3 ± 1.7% vs. 34.7 ± 22.2%; *p* < 0.05) and to the Prismaflex HF (16.6 ± 4.4% vs. 49.4 ± 19.5%; *p* < 0.01) hemofilters and significantly higher platelet loss as compared to the Prismaflex ST hemofilter (15.4 ± 3.0% vs. 9.5 ± 2.6%; *p* < 0.01).

In line, the multiFlux hemofilter also showed a lower β-TG increase over time as compared to the Ultraflux AV (134.3 ± 58.2 µg/L/h vs. 475.1 ± 408.8 µg/L/h; *p* < 0.05) and Prismaflex HF (231.4 ± 230.8 µg/L/h vs. 844.8 ± 935.8 µg/L/h; *p* = 0.051) hemofilters. For the Prismaflex ST hemofilter, β-TG data showed negative results, apparently attributable to the strong adsorptive characteristics of the AN69 ST membrane [31,32]. Therefore, these data were considered not valid for the analysis and therefore not reported.

#### 3.2.2. Plasmatic Coagulation Activation

For the characterization of plasmatic coagulation, the increase in thrombin–antithrombin III complex (TAT) was determined. Moreover, activated clotting time (ACT) was measured as an additional control parameter of the coagulation process. The results are summarized in Figure 5 and Supplementary Table S2.

TAT increase over time was at a comparable level between the multiFlux and Ultraflux AV hemofilters (5.7 ± 3.8 µg/L/h vs. 7.3 ± 4.7 µg/L/h; *p* = 0.07) and non-significantly lower vs. the Prismaflex HF hemofilter (6.6 ± 2.0 µg/L/h vs. 20.2 ± 20.5 µg/L/h; *p* = 0.07). Regarding the Prismaflex ST hemofilter, similar to β-TG, TAT data could not be analyzed, as no valid signal was measured due to the strong adsorption effects of the AN69 ST membrane [31,32].

ACT increased at the first measurement time point (15 min) for the multiFlux, Ultraflux AV and Prismaflex HF hemofilters, as compared to the pre values (0 min), in line with dilution effects and the initial platelet loss. The Prismaflex ST hemofilter showed a different pattern with a direct drop of the ACT, which is due to the strong heparin binding affinity by the AN69 ST membrane [33,34]. After 15 min, ACT decreased over time for all hemofilters, reflecting the ongoing coagulation process during blood recirculation. This decrease was lowest for the multiFlux hemofilter and reached statistical significance in the comparison vs. the Ultraflux AV (69.3 ± 127.9 s vs. 294.1 ± 188.0 s; *p* < 0.05) but not vs. the Prismaflex HF (145.6 ± 139.5 s vs. 270.9 ± 121.1 s; *p* = 0.13) and the Prismaflex ST (134.3 ± 125.0 s vs. 243.9 ± 85.0 s; *p* = 0.10) hemofilters.

#### 3.2.3. Leukocyte and Complement Activation

Leukocyte activation was assessed by measuring the increase in polymorphonuclear neutrophil (PMN) elastase, and complement activation was assessed by measuring the increase in complement factor sC5b-9. The results for both parameters are summarized in Figure 6 and Supplementary Table S2.

No significant differences were observed for PMN elastase increase, with slightly lower values for the Ultraflux AV hemofilter (31.5 ± 24.9 µg/L/h vs. 15.7 ± 11.2 µg/L/h; *p* = 0.11) and higher values for the Prismaflex HF hemofilter (51.5 ± 30.2 µg/L/h vs. 123.7 ± 198.0 µg/L/h; *p* = 0.18) as compared to the multiFlux hemofilter. Again, no valid signal was measured for the Prismaflex ST hemofilter due to the strong adsorption effects [31,32].

For sC5b-9, the multiFlux hemofilter showed a significantly lower increase over time as compared to the Ultraflux AV (738.7 ± 365.4 µg/L/h vs. 1145.7 ± 791.3 µg/L/h; *p* < 0.05), the Prismaflex HF (618.7 ± 515.6 µg/L/h vs. 1816.8 ± 1278.3 µg/L/h; *p* < 0.05) and the Prismaflex ST (982.7 ± 819.3 µg/L/h vs. 2681.8 ± 2080.1 µg/L/h; *p* < 0.05) hemofilters.

## 4. Discussion

In the present in vitro study, we investigated the novel multiFlux hemofilter intended for the treatment of patients with acute kidney injury requiring continuous kidney replacement therapy (CKRT). By comparing the new hemofilter to established hemofilters, we demonstrated that the multiFlux hemofilter exhibits strong performance and a favorable hemocompatibility profile in this in vitro setup.

Patients with acute kidney injury depend on safe and effective products for their treatment. The hemofilter is a key component of CKRT and largely determines treatment efficiency. Moreover, the large surface area of the hemofilter membrane comes with a substantial blood-material interface, resulting in potentially extensive interactions between the artificial surface and the patient’s blood, which may lead to hemocompatibility issues and adverse patient reactions [7,8,9,10,11].

Therefore, adequate preclinical evaluations are required for a new hemofilter. This is especially the case for performance and hemocompatibility testing. Although such in vitro studies cannot replace clinical studies, they can provide valuable insights into device performance and hemocompatibility and should therefore be designed to closely reflect the clinical setting, for example, by using blood or plasma instead of water for performance measurements or by applying dynamic rather than static conditions for hemocompatibility testing. Flow dynamics and the adsorption of plasma proteins to the membrane during treatment are major modulators of both performance and hemocompatibility and should therefore be considered in such investigations [21,35,36].

Protein adsorption to the membrane leads to the formation of a secondary layer that progressively alters the effective pore characteristics and tends to increase transport resistance. This phenomenon predominately affects the clearance of larger molecules, whereas the clearance of small molecules stays mainly stable [12,13,14,15,16,21,36]. This is in line with our current study, as the clearances of urea (60 Da) and creatinine (113 Da) remained largely stable over time, whereas the clearance for β2M (12 kDa) showed a clear decrease over time. Here, significant differences were observed between the four investigated hemofilters. The most stable performance was found for the multiFlux hemofilter, whereas the Ultraflux AV and Prismaflex HF hemofilters showed a comparable decrease in β2M clearance. In contrast, the Prismaflex ST hemofilter showed a different pattern compared to the other hemofilters with a generally low dialysate-side β2M clearance, i.e., a low appearance of β2M in the effluent. This low clearance is, however, partly attributed to the strong adsorptive characteristics of the AN69 ST membrane, leading to a lower β2M concentration in the effluent and subsequently lower dialysate-side clearance. Importantly, this lower dialysate-side clearance cannot be equated with lower overall β2M removal, as adsorption effects also contribute to the total solute removal. Therefore, critical interpretation of results from such studies is essential, especially when comparing membranes with strong adsorptive characteristics.

Protein adsorption into the membrane also influences hemocompatibility of the hemofilter. Upon adsorption to the membrane, proteins undergo conformational changes or denaturation, thereby activating various biological pathways in the patient’s blood, including platelet activation, coagulation, complement activation and immune cell activation [7,11,17,18,23,29,35,37,38]. Excessive activation of these pathways may contribute to the inflammatory burden of the patients and to thrombotic complications during extracorporeal treatment. Therefore, minimizing such blood–membrane interactions is a major objective in the development of novel hemofilter membranes.

The extent and composition of protein adsorption are strongly influenced by the membrane properties, with membrane hydrophilicity being a main determinant. For poly(ether)sulfone-based membranes, typically, the co-polymer polyvinylpyrrolidone (PVP) is used to increase hydrophilicity due to the hydrophobic nature of poly(ether)sulfone [39]. Experimental studies have shown that increased membrane hydrophilicity improves hemocompatibility and performance stability due to lower membrane fouling [12,21,22,23,24,25,26,27,28,29].

The manufacturing process of the multiFlux membrane (Helixone *hydro*) was refined to increase the PVP content on the blood side of the membrane. To achieve this, PVP is added to the precipitation fluid, in addition to its incorporation into the spinning solution during the standard manufacturing process. Previous experimental studies investigated the Helixone *hydro* membrane in terms of PVP content, hydrophilicity and membrane fouling [21,28,30,40]. Using X-ray photoelectron spectroscopy, it was shown that the Helixone *hydro* membrane had a higher PVP content on the blood-side surface as compared to the other investigated polysulfone- and polyethersulfone-based membranes [28]. Moreover, contact-angle and atomic force microscopy measurements demonstrated that the Helixone *hydro* membrane is more hydrophilic than a membrane with lower PVP content [21,40]. These membrane characteristics were linked to reduced membrane fouling [21,30,40], which is consistent with findings from studies on experimental membranes showing a direct relationship between increased PVP content, enhanced hydrophilicity and reduced protein adsorption [23,24].

In the present study we showed that the multiFlux hemofilter, with its more hydrophilic membrane, is associated with improved performance stability for β2M clearance and a favorable hemocompatibility profile, especially for platelet and complement activation, which is in line with previous reports on membrane hydrophilicity [12,21,22,23,24,25,26,27,28,29]. Advantages in sustained performance were observed for β2M, as protein adsorption mainly affects the clearance of larger molecules. For the small molecules urea and creatinine, all hemofilters achieved broadly comparable clearances, which can mainly be attributed to the low flow conditions applied in this study to realistically mimic clinical treatment conditions. Thus, the similar clearance values for these small molecules primarily reflect the operational limitation resulting from the effluent flow rates, rather than an absence of differences in membrane permeability. Also regarding hemocompatibility, the multiFlux hemofilter showed a favorable profile, especially in terms of platelet activation (vs. Ultraflux AV and Prismaflex HF) and complement activation (vs. all comparators). Similar to the β2M clearance measurements, the hemocompatibility results obtained for the Prismaflex ST hemofilter should be interpreted with care due to the strong adsorption effects of the AN69 ST membrane. Several hemocompatibility parameters could not be analyzed (β-TG, TAT, and PMN elastase), while others showed a different pattern (ACT) than the other investigated hemofilters.

It is important to note that the present study was conducted in vitro and, therefore, no direct clinical conclusions can be drawn. This study used blood from healthy donors and bovine plasma to assess hemocompatibility and performance, which cannot fully represent the complex clinical situation of patients with acute kidney injury, including ongoing systemic inflammation, concomitant medication, or interactions of blood cells with the endothelium following cellular activation. Nevertheless, such in vitro studies are essential for providing initial information on the performance and hemocompatibility of a new product before it is applied in patients. A prospective clinical study is currently planned to evaluate whether the benefits observed in this in vitro study will also translate into better clinical results [41]. This study will investigate performance parameters, including urea and creatinine clearance, as well as patient safety and hemofilter life span.

In summary, the present in vitro study investigated, for the first time, the novel multiFlux hemofilter. The hemofilter demonstrated the highest in vitro middle-molecule clearance and a favorable hemocompatibility profile, particularly with regard to platelet and complement activation. These results support previous findings that increased membrane hydrophilicity is associated with improved performance stability and hemocompatibility of dialysis membranes.

## Figures and Tables

**Figure 1:**
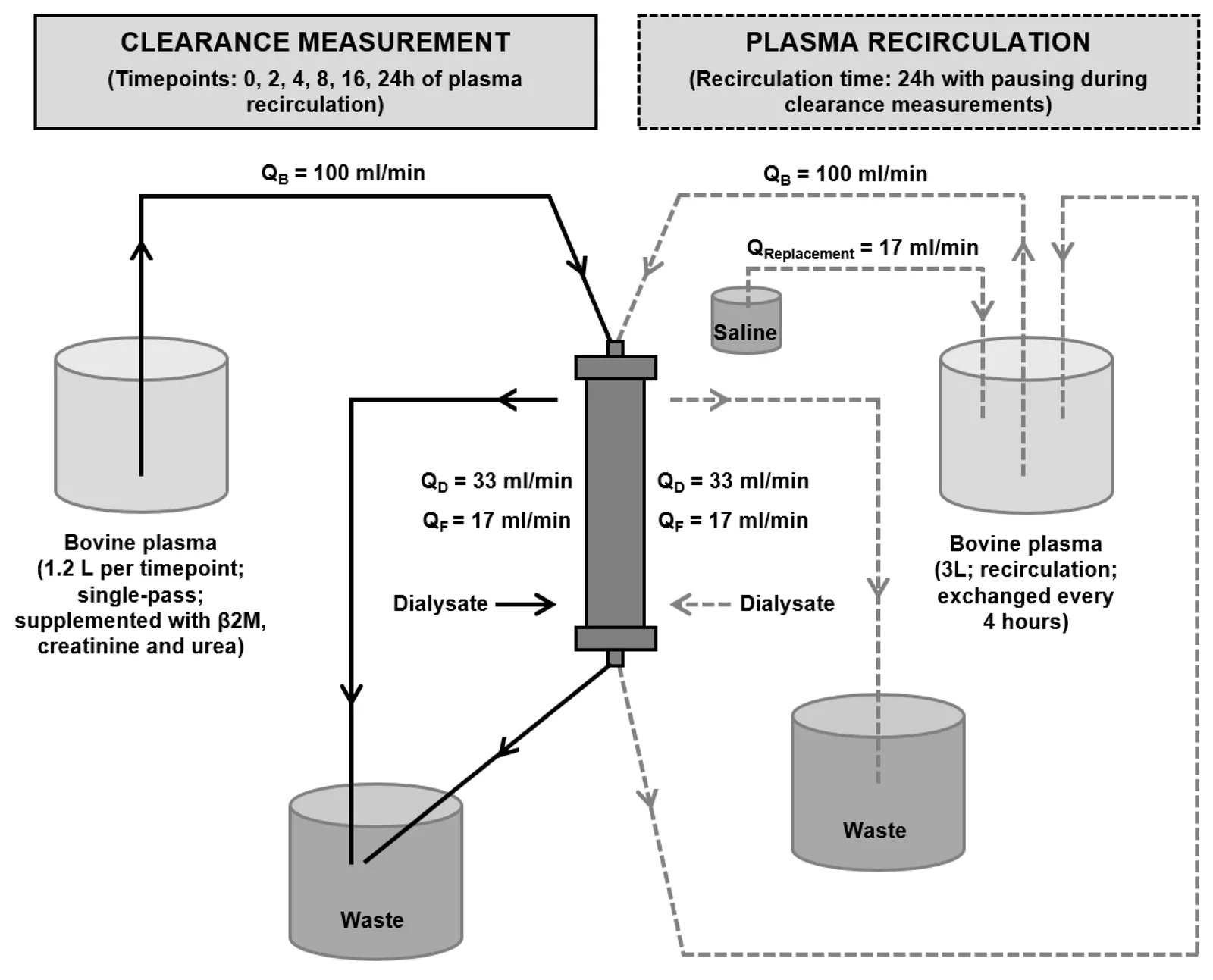
Schematic illustration of the setup to determine performance over time. The left part shows the determination of urea, creatinine and β2-microglobulin clearance at the following time points: 0, 2, 4, 8, 16, and 24 h. The right part shows the plasma recirculation to induce membrane fouling over 24 h. For each hemofilter, three independent experiments were performed.

**Figure 2:**
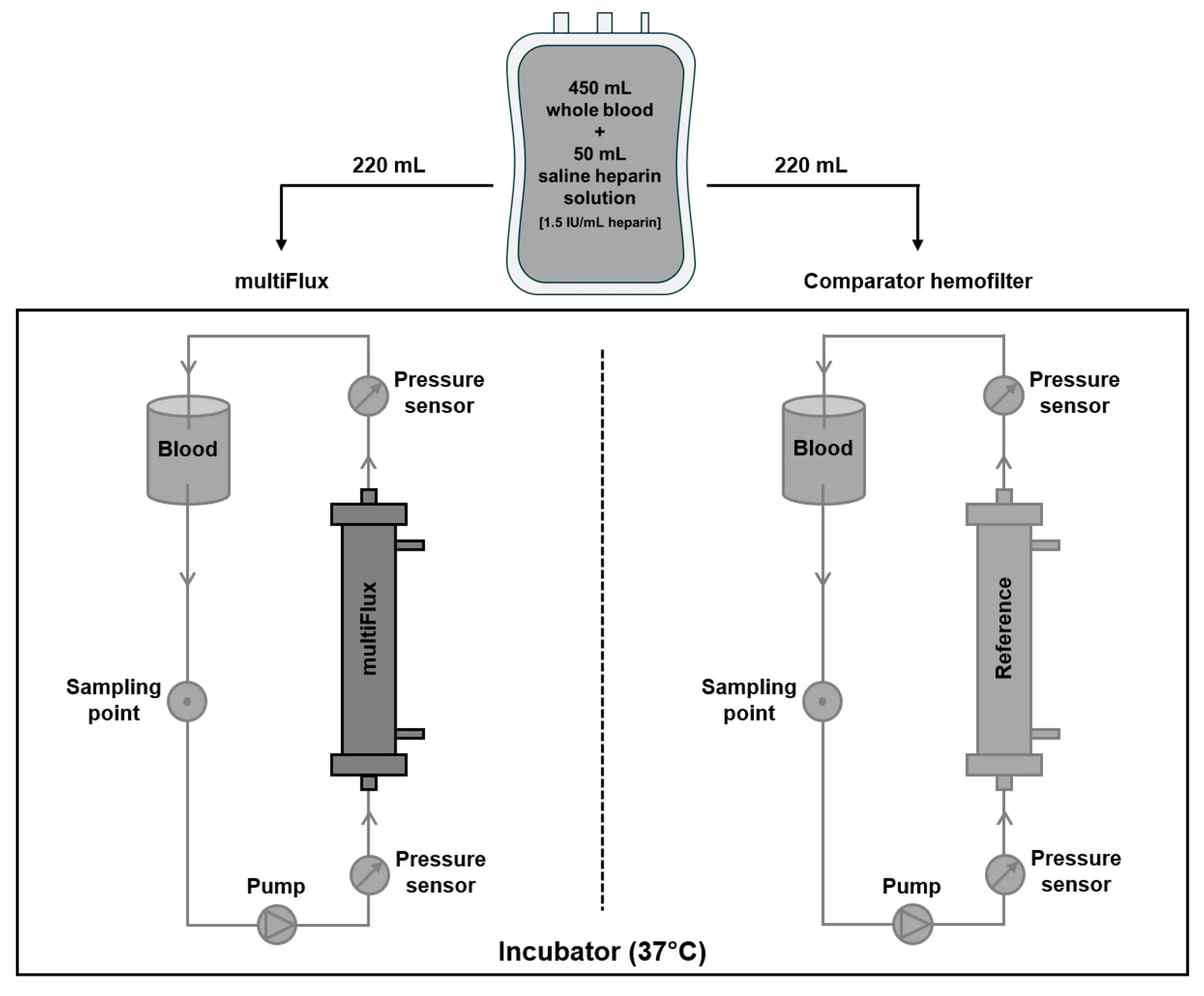
Schematic illustration of the blood recirculation setup to characterize the hemocompatibility of hemofilters. For each experiment, blood from the same donor was split to directly compare the multiFlux with a comparator hemofilter to reduce inter-donor variability. Blood samples were taken before recirculation (0 min) and at different recirculation time points (15, 30, 60, 120 and 180 min) to measure the respective hemocompatibility makers. Six independent experiments were performed for each pairwise comparison. Thus, a total of 18 blood donations were required for the three comparisons (comparison 1: multiFlux vs. Ultraflux AV, *n* = 6; comparison 2: multiFlux vs. Prismaflex HF, *n* = 6; comparison 3: multiFlux vs. Prismaflex ST, *n* = 6).

**Figure 3:**
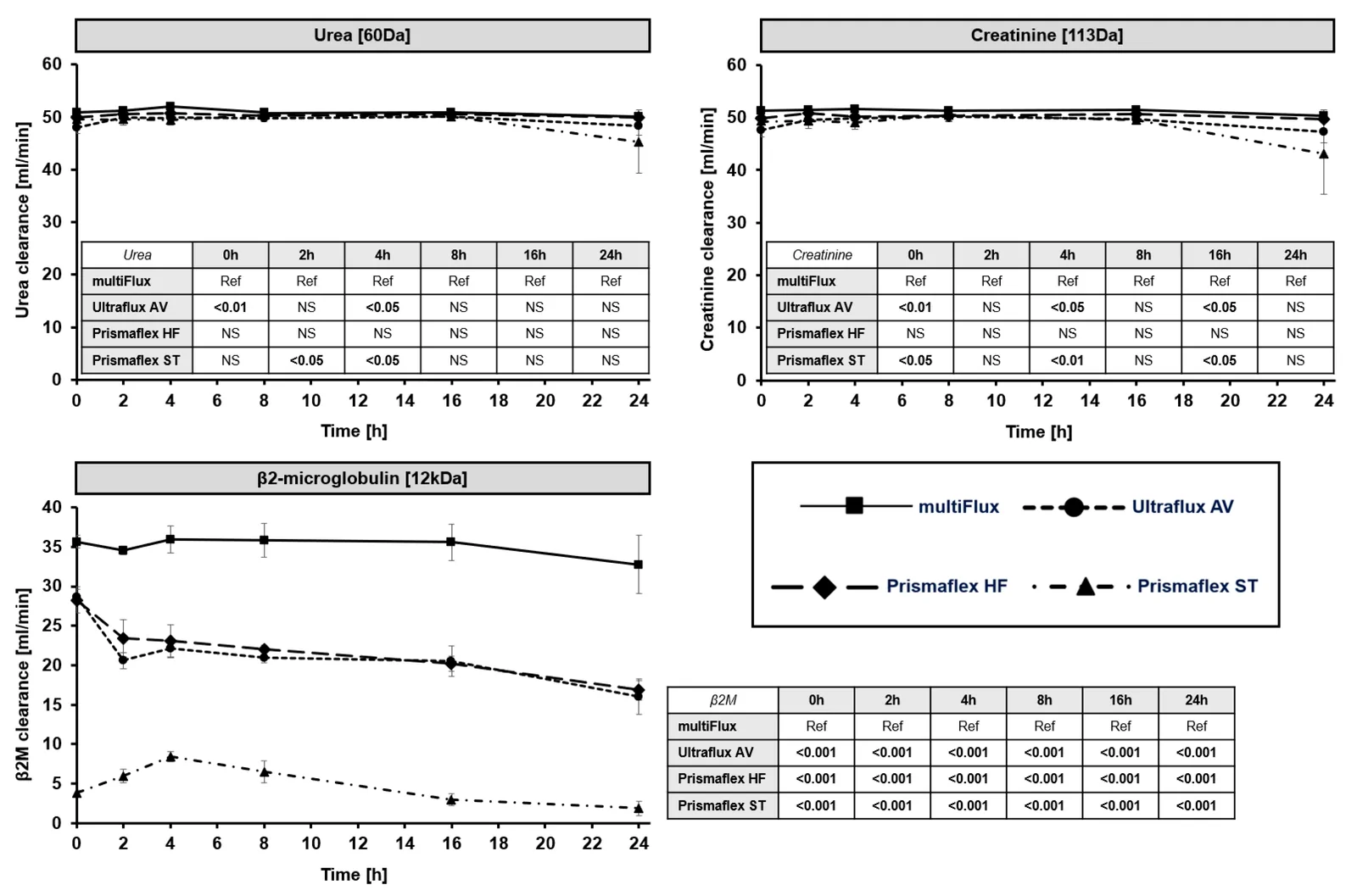
Hemofilter performance over time. At various time points (0, 2, 4, 8, 16, and 24 h) of plasma recirculation, dialysate-side plasma clearances of urea, creatinine and β2M were determined for the four hemofilters (multiFlux 160, Ultraflux AV 1000 S, Prismaflex HF1400 and Prismaflex ST150). Means ± standard deviations of three independent experiments per time point are shown for each hemofilter. One-way ANOVA with Dunnett’s post hoc test was applied to compare the multiFlux hemofilter to the other hemofilters at all time points. The respective *p* values are shown in the tables within the figure for each parameter and time point. Ref: Reference; NS: not significant.

**Figure 4:**
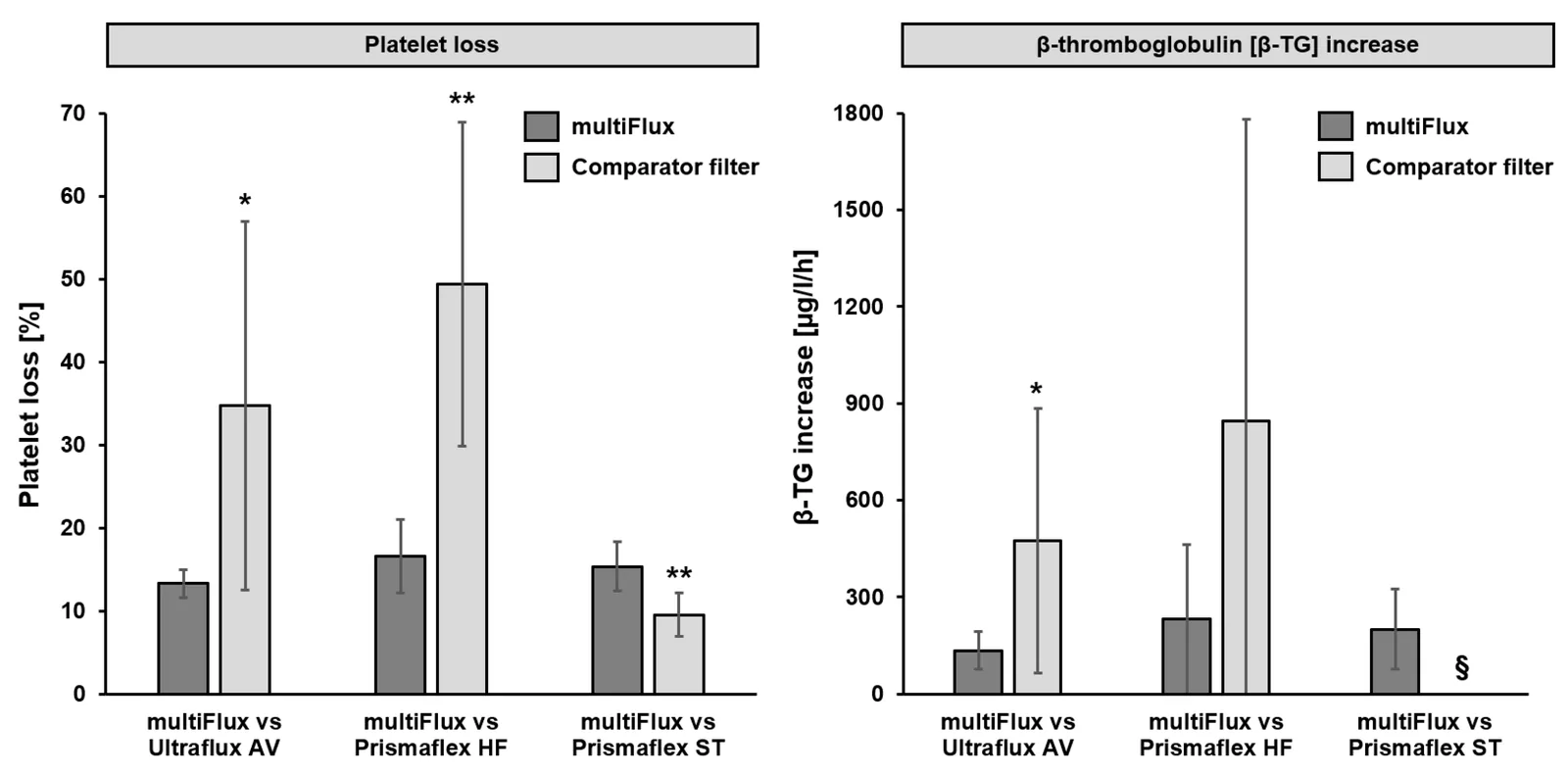
Platelet loss and β-TG increase over time. Platelet loss (**left**) and β-TG increase per hour (**right**) were determined in pairwise comparisons of the multiFlux 130 vs. three comparator hemofilters (Ultraflux AV 600 S, Prismaflex HF1400 and Prismaflex ST150). Means ± standard deviations of six independent experiments are shown for each comparison. In case standard deviations exceed the mean, the part below the *x*-axis is not shown. The reported level of statistical significance is indicated in the pairwise comparisons with the multiFlux hemofilter (paired Student’s *t*-test, * *p* < 0.05; ** *p* < 0.01). § For the Prismaflex ST hemofilter, no valid signal could be observed over time, preventing analysis of this marker.

**Figure 5:**
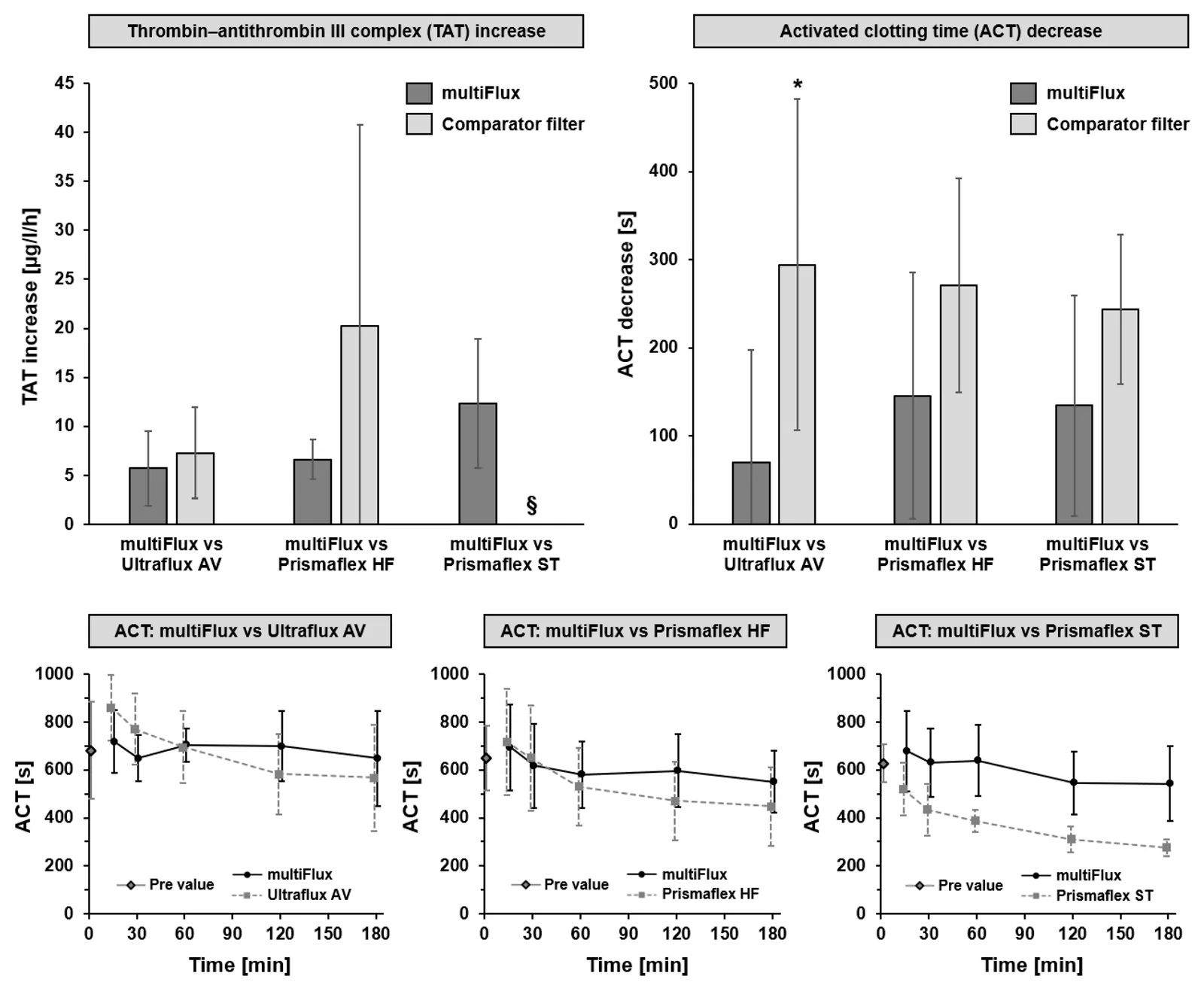
Thrombin–antithrombin III complex (TAT) increase and activated clotting time (ACT) over time. TAT increase per hour (upper left part) and ACT (upper right part and lower part) were determined in pairwise comparisons of the multiFlux 130 vs. three comparator hemofilters (Ultraflux AV 600 S, Prismaflex HF1400 and Prismaflex ST150). ACT decrease was determined between 15 and 180 min of blood recirculation. Means ± standard deviations of six independent experiments are shown for each comparison. In case standard deviations exceed the mean, the part below the *x*-axis is not shown. The reported level of statistical significance is indicated in the pairwise comparisons with the multiFlux hemofilter (paired Student’s *t*-test, * *p* < 0.05). § For the Prismaflex ST hemofilter, no valid signal could be observed over time, preventing analysis of this marker.

**Figure 6:**
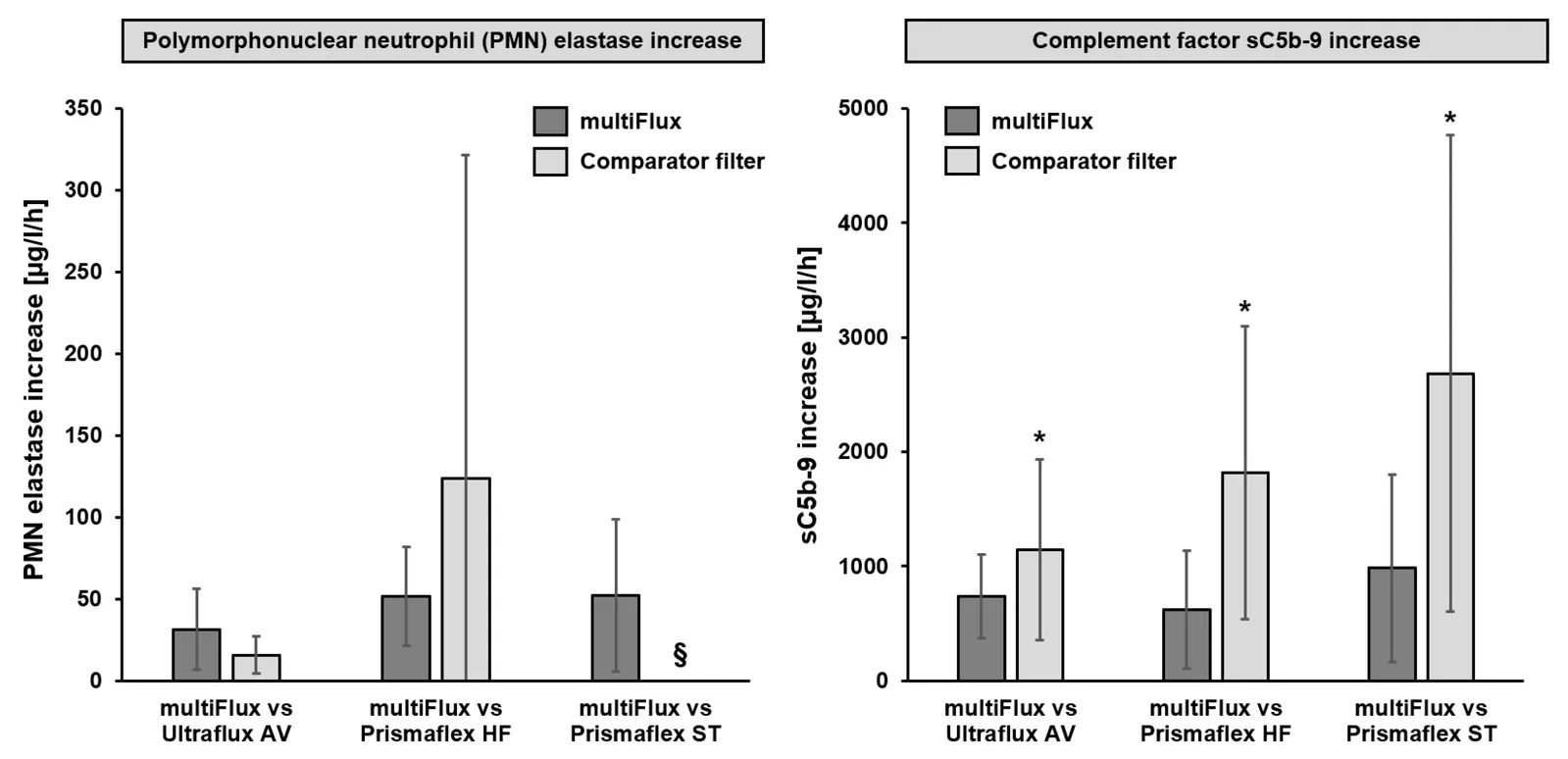
Polymorphonuclear neutrophil (PMN) elastase and complement factor sC5b-9 increase over time. PMN elastase (**left**) and sC5b-9 increase per hour (**right**) were determined in pairwise comparisons of the multiFlux 130 vs. three comparator hemofilters (Ultraflux AV 600 S, Prismaflex HF1400 and Prismaflex ST150). Means ± standard deviations of six independent experiments are shown for each comparison. In case standard deviations exceed the mean, the part below the *x*-axis is not shown. The reported level of statistical significance is indicated in the pairwise comparisons with the multiFlux hemofilter (paired Student’s *t*-test, * *p* < 0.05). § For the Prismaflex ST hemofilter, no valid signal could be observed over time, preventing analysis of this marker.

**Table 1:** Investigated hemofilters in the present study.

Hemofilter	Manufacturer	Membrane Name	Membrane Material	Sterilization	Membrane Surface [m^2^]	Fiber Inner Diameter [µm]
multiFlux 130	Fresenius Medical Care	Helixone *hydro*	Polysulfone, PVP	INLINE steam	1.3	210
Ultraflux AV 600 S	Fresenius Medical Care	Fresenius Polysulfone^®^	Polysulfone, PVP	INLINE steam	1.4	220
Prismaflex HF1400	Baxter/Vantive	N/A	PAES, PVP	EtO	1.4	215
Prismaflex ST150	Baxter/Vantive	N/A	AN69 ST	EtO	1.5	240

PVP: Polyvinylpyrrolidone; PAES: Polyarylethersulfone; AN69 ST: Acrylonitrile and sodium methallyl sulfonate copolymer + polyethylene imine (surface treatment agent); EtO: Ethylene oxide; N/A: not available; hemofilter models displayed for hemocompatibility testing; for performance experiments, multiFlux 160 (1.6 m^2^) and Ultraflux AV 1000 S (1.8 m^2^) were used.

## Data Availability

The original contributions presented in this study are included in the article/Supplementary Materials. Further inquiries can be directed to the corresponding author.

## Supplementary Materials

The following supporting information can be downloaded at: https://www.mdpi.com/article/10.3390/membranes16100338/s1, Figure S1 and Tables S1 and S2. Figure S1. Surface-normalized β2-microglobulin clearance over time. At various time points (0, 2, 4, 8, 16 and 24 h) of plasma recirculation, dialysate-side plasma clearance of β2M was determined for the four hemofilters (multiFlux 160, Ultraflux AV 1000 S, Prismaflex HF1400 and Prismaflex ST150). Clearance values were surface-normalized. Means ± standard deviations of three independent experiments per time point are shown for each hemofilter. One-way ANOVA with Dunnett’s post hoc test was applied to compare the multiFlux to the other hemofilters at all time points. The respective *p* values are shown in the tables within the figure for each parameter and time point. Table S1. Performance data. Table S2. Hemocompatibility data.

## Abbreviations

The following abbreviations are used in this manuscript:

ACT	Activated clotting time
AN69 ST	Acrylonitrile and sodium methallyl sulfonate copolymer + polyethylene imine (surface treatment agent)
AUC	Area under the curve
β2M	β2-microglobulin
β-TG	β-thromboglobulin
CKRT	Continuous kidney replacement therapy
ELISA	Enzyme-linked immunosorbent assay
EtO	Ethylene oxide
N/A	Not available
PAES	Polyarylethersulfone
PMN	Polymorphonuclear neutrophil
PVP	Polyvinylpyrrolidone
Q_B_	Blood-side (blood/plasma) flow rate
Q_D_	Dialysate flow rate
Q_F_	Filtrate flow rate
TAT	Thrombin–antithrombin III complex
