# Supplementary material for: Characterization of In Vitro Performance and Hemocompatibility of a Novel Hemofilter for Continuous Kidney Replacement Therapy
Source: Membranes (Basel). 2026 Oct 12;16(10):338. doi: 10.3390/membranes16100338 (PMC13707138; doi:10.3390/membranes16100338)
Supplement: Supplementary file 1 [file membranes-16-00338-s001.zip › membranes-4588161-supplementary.pdf]

Supplementary material

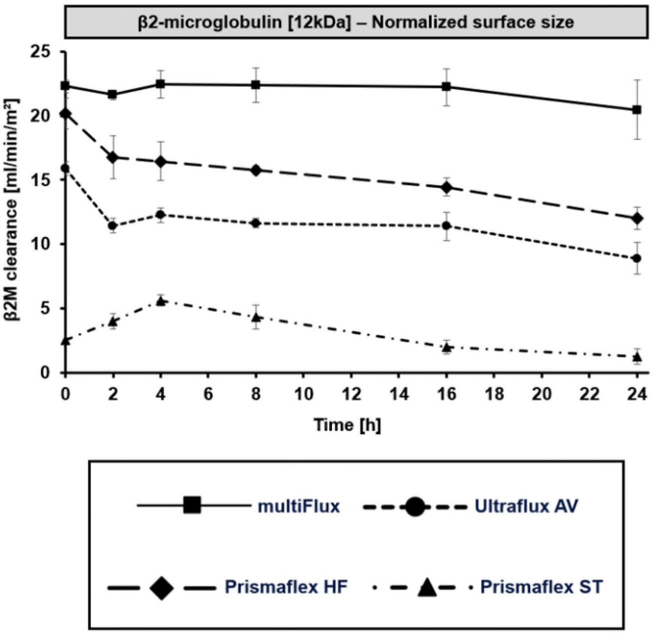

β2M	0h	2h	4h	8h	16h	24h
multiFlux	Ref	Ref	Ref	Ref	Ref	Ref
Ultraflux AV	<0.001	<0.001	<0.001	<0.001	<0.001	<0.001
Prismaflex HF	<0.05	<0.01	<0.001	<0.001	<0.001	<0.001
Prismaflex ST	<0.001	<0.001	<0.001	<0.001	<0.001	<0.001

Figure S1. Surface-normalized β2-microglobulin clearance over time. At various time points (0, 2, 4, 8, 16 and 24 h) of plasma recirculation, dialysate-side plasma clearance of β2M was determined for the four hemofilters (multiFlux 160, Ultraflux AV 1000 S, Prismaflex HF1400 and Prismaflex ST150). Clearance values were surface-normalized. Means ± standard deviations of three independent experiments per time point are shown for each hemofilter. One-way ANOVA with Dunnett's post hoc test was applied to compare the multiFlux to the other hemofilters at all time points. The respective p values are shown in the tables within the figure for each parameter and time point.

Table S1. Performance data.

		0h	2h	4h	8h	16h	24h
				Urea			
multiFlux	**Mean**	50.9	51.2	51.9	50.8	50.9	50.1
	**SD**	0.3	0.2	0.7	0.2	0.2	0.7
Ultraflux AV	**Mean**	48.0	50.0	49.8	49.8	50.0	48.4
	**SD**	1.2	0.3	0.7	0.7	0.8	1.8
	**P (vs multiFlux)**	**0.004**	0.135	**0.027**	0.065	0.250	0.829
Prismaflex HF	**Mean**	49.9	50.6	50.7	50.3	50.6	49.9
	**SD**	0.7	0.7	0.6	0.4	0.3	0.4
	**P (vs multiFlux)**	0.276	0.585	0.180	0.419	0.867	1.000
Prismaflex ST	**Mean**	49.6	49.6	49.5	50.3	50.0	45.3
	**SD**	0.6	1.1	1.0	0.6	0.8	6.0
	**P (vs multiFlux)**	0.134	**0.041**	**0.011**	0.504	0.237	0.217
				Creatinine			
multiFlux	**Mean**	51.3	51.4	51.6	51.3	51.4	50.3
	**SD**	0.4	0.1	0.4	0.8	0.2	1.2
Ultraflux AV	**Mean**	47.6	49.6	49.9	50.1	49.6	47.3
	**SD**	1.2	0.5	0.5	0.9	0.6	2.0
	**P (vs multiFlux)**	**0.001**	0.124	**0.046**	0.323	**0.019**	0.692

**Prismaflex HF**	**Mean**	49.8	50.8	50.1	50.3	50.7	49.8
	**SD**	0.5	1.4	0.6	0.6	1.0	0.3
	**P (vs multiFlux)**	0.086	0.807	0.081	0.443	0.381	0.997
**Prismaflex ST**	**Mean**	49.3	49.3	49.0	50.6	49.5	43.1
	**SD**	0.4	1.3	1.2	1.2	0.3	7.6
	**P (vs multiFlux)**	**0.024**	0.079	**0.006**	0.645	**0.011**	0.135
				β2-microglobulin			
**multiFlux**	**Mean**	35.7	34.6	35.9	35.8	35.6	32.8
	**SD**	0.8	0.5	1.7	2.1	2.3	3.7
**Ultraflux AV**	**Mean**	28.6	20.6	22.1	21.0	20.5	16.0
	**SD**	1.0	1.0	1.0	0.6	2.0	2.2
	**P (vs multiFlux)**	**<0.001**	**<0.001**	**<0.001**	**<0.001**	**<0.001**	**<0.001**
**Prismaflex HF**	**Mean**	28.3	23.5	23.1	22.1	20.2	16.8
	**SD**	1.7	2.3	2.1	0.5	1.0	1.2
	**P (vs multiFlux)**	**<0.001**	**<0.001**	**<0.001**	**<0.001**	**<0.001**	**<0.001**
**Prismaflex ST**	**Mean**	3.8	6.0	8.4	6.5	3.0	1.9
	**SD**	0.3	0.9	0.7	1.4	0.8	0.9
	**P (vs multiFlux)**	**<0.001**	**<0.001**	**<0.001**	**<0.001**	**<0.001**	**<0.001**

Table S2. Hemocompatibility data.

Category	Parameter	Comparison 1: multiFlux vs. Ultraflux AV	Comparison 2: multiFlux vs. Prismaflex HF	Comparison 3: multiFlux vs. Prismaflex ST
Platelet activation	Platelet loss	13.3±1.7% vs. 34.7±22.2% **p<0.05**	16.6±4.4% vs. 49.4±19.5% **p<0.01**	15.4±3.0% vs. 9.5±2.6% **p<0.01**
	β-TG increase	134.3±58.2 µg/l/h vs. 475.1±408.8 µg/l/h **p<0.05**	231.4±230.8 µg/l/h vs. 844.8±935.8 µg/l/h p=0.051	Data not valid for Prismaflex ST
Coagulation activation	TAT increase	5.7±3.8 µg/l/h vs. 7.3±4.7 µg/l/h p=0.07	6.6±2.0 vs. 20.2±20.5 µg/l/h p=0.07	Data not valid for Prismaflex ST
	ACT decrease	69.3±127.9s vs. 294.1±188.0s **p<0.05**	145.6±139.5s vs. 270.9±121.1s p=0.13	134.3±125.0s vs. 243.9±85.0s p=0.10
Leukocyte activation	PMN elastase increase	31.5±24.9 vs. 15.7±11.2 µg/l/h p=0.11	51.5±30.2 vs. 123.7±198.0 µg/l/h p=0.18	Data not valid for Prismaflex ST
Complement activation	sC5b-9 increase	738.7±365.4 µg/l/h vs. 1145.7±791.3 µg/l/h **p<0.05**	618.7±515.6 µg/l/h vs. 1816.8±1278.3 µg/l/h **p<0.05**	982.7±819.3 µg/l/h vs. 2681.8±2080.1 µg/l/h **p<0.05**
